# Summary of the National Advisory Committee on Immunization (NACI) Statement: Updated guidance to protect infants and children from respiratory syncytial virus disease

**DOI:** 10.14745/ccdr.v52i06a01

**Published:** 2026-06-30

**Authors:** April Killikelly, Winnie Siu, Nicholas Brousseau

**Affiliations:** 1 Centre for Immunization Surveillance and Programs, Public Health Agency of Canada, Ottawa, ON; 2 School of Epidemiology and Public Health, Faculty of Medicine, University of Ottawa, Ottawa, ON; 3 Département de médecine sociale et préventive, Faculté de médecine, Université Laval, Québec, QC

**Keywords:** immunization, vaccination, monoclonal antibodies, respiratory syncytial virus, infants, pregnancy

## Abstract

**Background:**

Respiratory syncytial virus (RSV) is a leading cause of hospitalization among Canadian infants. In March 2026, the National Advisory Committee on Immunization (NACI) released updated guidance reflecting new evidence on the monoclonal antibodies (mAbs) nirsevimab (Beyfortus®) and clesrovimab (Enflosia) and the RSVpreF pregnancy vaccine (Abrysvo). This article summarizes NACI’s updated guidance on protecting infants and children from severe RSV disease.

**Methods:**

To develop the statement, NACI used its standard evidence-based process, reviewing clinical trial data, real world effectiveness studies, RSV epidemiology, and Canadian cost utility modelling, as well as ethics, equity, feasibility and acceptability considerations, to inform program recommendations.

**Results:**

Both mAbs and the RSVpreF vaccine demonstrated strong protection against RSV related hospitalization, intensive care unit (ICU) admission, and medically attended illness in infants. All products showed favourable safety profiles. The RSVpreF vaccine was effective when administered ≥14 days prior to delivery. Economic modelling indicated that a combined program of RSVpreF vaccination during pregnancy and targeted infant mAb administration offers the best value for money at current product prices. NACI strongly recommends universal seasonal RSV immunization for infants. Jurisdictions may choose either 1) a universal infant mAb program or 2) a combined pregnancy RSVpreF vaccination plus targeted infant mAb administration program. Infants at increased risk of severe RSV disease should receive mAbs regardless of gestational RSVpreF vaccination status.

**Conclusion:**

Universal seasonal RSV immunization will substantially reduce RSV associated morbidity in infants and could likely be achieved through both program approaches. At current product list prices, the combined pregnancy-infant program offers better value than a universal mAb program. Jurisdictions should consider local epidemiology, feasibility, cost-effectiveness considerations and equity when selecting a program model.

## Introduction

Respiratory syncytial virus (RSV) is one of the most common respiratory pathogens affecting infants and young children. Nearly all children acquire RSV by two years of age, with the highest risk of severe disease—including hospitalization and intensive care unit (ICU) admission—occurring during the first months of life. While chronic lung disease, congenital heart disease, prematurity, immunodeficiency, and certain genetic conditions increase risk, most infants hospitalized with RSV each year are otherwise healthy.

In 2026, the authorization of clesrovimab in Canada, alongside expanding real-world evidence for the previously authorized products nirsevimab and RSVpreF, prompted the National Advisory Committee on Immunization (NACI) to review and update its previous guidance to support national program planning (1).

## Methods

NACI followed its established evidence-based process, which included:

• Reviewing randomized controlled trial data for monoclonal antibodies (mAbs) and RSVpreF.

• Examining real-world vaccine and mAb effectiveness from multiple countries, including new Canadian evidence.

• Assessing RSV epidemiology in infants and in pregnancy.

• Applying the Ethics, Equity, Feasibility, and Acceptability (EEFA) framework.

• Evaluating cost-effectiveness using an updated Canadian cost-utility model incorporating product effectiveness, protection duration, hospitalization rates, and current product list prices (2).

Recommendations were formulated through consensus of the RSV Working Group and approved by the full NACI committee.

## Results

Clinical trials and real-world evaluations consistently demonstrate that the long-acting mAbs, nirsevimab and clesrovimab, and the RSVpreF pregnancy vaccine, significantly reduce medically attended RSV illness, hospitalization, and ICU admission in infants (1). Clesrovimab showed 84% efficacy against RSV hospitalization with no ICU admissions in the pivotal trial. Nirsevimab demonstrated strong real-world effectiveness, lowering hospitalization and ICU admission rates across multiple jurisdictions. The RSVpreF vaccination in pregnancy reduced RSV-associated hospitalization and severe disease in infants when administered at least 14 days prior to delivery, with the highest protective effects observed during the first three months of life.

All products demonstrated favourable safety profiles. Clinical trials found no meaningful differences in severe adverse events compared with placebo or the previously used short-acting mAb palivizumab. Surveillance data did not identify significant safety concerns with nirsevimab. For RSVpreF, an imbalance in preterm births was observed in a clinical trial between individuals who received RSVpreF during pregnancy and those who did not (3). Recent real-world evidence from high-income settings did not show an increased risk of preterm birth. Monitoring for preterm birth and for other obstetric outcomes such as hypertensive disorders continues.

Economic analyses showed that universal programs are not cost-effective at current list prices. Among broader programs, the most economically favourable strategy is a combined program of RSVpreF vaccination during pregnancy for infants expected to be born during the RSV season, plus targeted mAb administration for infants at high risk. For healthy infants born before the RSV season, cost-effectiveness declines with increasing age at season start, and jurisdictions may consider prioritizing the youngest of these infants.

## Discussion

### Key messages

Several key messages emerge from the review of evidence:

• Respiratory syncytial virus remains a leading cause of hospitalization in Canadian infants, particularly in those younger than six months of age.

• New evidence on long-acting mAbs (nirsevimab, clesrovimab) and the RSVpreF pregnancy vaccine supports new protection strategies for infants.

• Either an infant mAb program or a combined pregnancy vaccine with a targeted infant mAb program could be used for universal seasonal RSV immunization programs. Infants at increased risk for severe RSV disease should receive mAbs regardless of gestational vaccination status.

• Economic modelling indicates that a combined pregnancy RSVpreF and targeted infant mAb program is the most economically favourable option at current list prices.

### Summary of recommendations

NACI strongly recommends that provinces and territories implement universal seasonal RSV immunization for infants, delivered through one of two approaches, with an additional recommendation for infants at increased risk of RSV disease:

**Recommendation 1:** NACI recommends to implement universal seasonal RSV immunization for infants. **(*Strong NACI recommendation*)**

**Recommendation 2:** NACI recommends that jurisdictions implement a seasonal RSV immunization program, based on local context, feasibility and program priorities:

• Universal Infant Monoclonal Antibody Program

o Administer nirsevimab or clesrovimab to all infants entering their first RSV season, with second-season doses for children at continued increased risk.

OR

• Combined Pregnancy + Targeted Infant Program

o Offer RSVpreF vaccination during pregnancy (28–36 weeks gestation) for infants expected to be born during RSV season.

**Note:** NACI recommends RSVpreF vaccine can be given during pregnancy from 28–36 weeks gestation. The RSVpreF vaccine is authorized in Canada at 32–36 weeks gestation. The off-label recommendation from NACI is supported by safety and efficacy data, supports broader access and opportunities for immunization, and aligns with recommendations by the World Health Organization.

o Offer mAbs to:

▪ Infants at increased risk of severe RSV disease

▪ Infants born <14 days after maternal vaccination

▪ Infants born to unvaccinated individuals

(*Strong NACI recommendation*)

**Recommendation 3:** Infants at increased risk of severe RSV disease

• Infants at increased medical risk of severe RSV disease (**List 1**) should receive mAbs in both their first and second RSV seasons. Infants for whom transportation for severe RSV disease treatment is complex, and/or whose risk of severe RSV disease intersects with established social and structural health determinants (such as those experienced by some individuals in or from First Nations, Inuit and Métis communities) are also at increased risk and should receive a mAb in their first RSV season.

**Note:** Clesrovimab is not authorized for infants and children at ongoing risk in their second RSV season but could be considered off-label based on evidence of immunogenicity and safety.

(*Strong NACI recommendation*)

**List 1: Infants and children at increased medical risk of severe respiratory syncytial virus disease**

**Table:** 

Infants and children at increased medical risk of severe RSV disease are those with: • Chronic lung disease, including bronchopulmonary dysplasia, requiring ongoing assisted ventilation, oxygen therapy or chronic medical therapy in the six months prior to the start of the RSV season • Cystic fibrosis with respiratory involvement and/or growth delay • Haemodynamically significant chronic cardiac disease • Severe immunodeficiency • Severe congenital airway anomalies impairing clearing of respiratory secretions • Neuromuscular disease impairing clearing of respiratory secretions • Down syndrome Premature infants less than 32 weeks gestational age are also at increased medical risk of severe RSV disease.

Abbreviation: RSV, respiratory syncytial virus

### Limitations

These recommendations do not include individual-level recommendations as it is anticipated that immunizations will be primarily offered through public programs.

## Conclusion

NACI recommends that universal seasonal RSV immunization programs be implemented, and that either a program of mAbs for all infants, or the pregnancy vaccination combined with targeted mAb administration, can be used. At present, the combined approach of RSVpreF vaccination during pregnancy and targeted mAbs for infants at high risk offers the best value for money among universal program options while ensuring that infants at increased risk receive optimal protection.

Jurisdictions are encouraged to apply local epidemiology, feasibility, cost considerations, and equity principles when implementing RSV prevention programs to protect infants across Canada.
